# Incidence of Intracranial Melanoma Progression in the Setting of Positive Extracranial Response to Targeted Therapy and Immunotherapy: An Indication for More Frequent Screening in This Population?

**DOI:** 10.7759/cureus.13648

**Published:** 2021-03-02

**Authors:** Domenico A Gattozzi, Casey Rosso, Bryan A Schatmeyer, Jean-Luc K Kabangu, Gary C Doolittle, Fen Wang, Timothy Stepp

**Affiliations:** 1 Neurosurgery, University of Kansas Medical Center, Kansas City, USA; 2 Neurosurgery, University of Kansas Medical School, Kansas City, USA; 3 Hematology/Oncology, University of Kansas Medical Center, Kansas City, USA; 4 Radiation Oncology, University of Kansas Medical Center, Kansas City, USA

**Keywords:** melanoma, intracranial, metastatic melanoma, metastases, stereotactic radiosurgery, immunotherapy, pembrolizumab, nivolumab, ipilimumab

## Abstract

Background and objective

The incidence of intracranial metastases from melanoma is on the rise. In this study, we aimed to determine the incidence of intracranial disease progression in patients on BRAF/MEK targeted therapy and immunotherapy in the setting of controlled or improving extracranial disease.

Methods

This was a single-center, retrospective review that involved patients who underwent stereotactic radiosurgery (SRS) for intracranial metastatic melanoma between January 1, 2014, and December 31, 2018. We focused on BRAF/MEK mutation status and dates of treatment with BRAF/MEK targeted therapy, immunotherapy [ipilimumab (Yervoy), nivolumab (Opdivo), or pembrolizumab (Keytruda)], and combination targeted and immunotherapy.

Results

A total of 51 patients were enrolled: 36 males and 15 females. The average age of the patients was 58.6 years, and 26 among them were BRAF mutation-positive. Seventeen had prior surgery with SRS as adjuvant therapy. The other 34 had SRS as primary treatment. Forty-two patients had extracranial disease present at the time of SRS. There were 34 patients treated with targeted and immune therapy. Overall, 16 patients (47.1%) demonstrated controlled or improving extracranial disease, and 18 (52.9%) demonstrated progressing extracranial disease at the time of SRS. In the subgroup analysis, patients treated with BRAF/MEK targeted therapy demonstrated a 75% rate of extracranial disease control. The extracranial disease was controlled in 43.75% of patients on immunotherapy with intracranial progression, while it was controlled in 30% of patients on both BRAF/MEK targeted therapy and immunotherapy with intracranial progression. Sixteen patients (47.1%) developed intracranial metastasis in our study while having a stable systemic disease with BRAF/MEK targeted therapy, immunotherapy, or a combination of the two.

Conclusion

Based on our findings, a systemic response to targeted therapy and immunotherapy does not necessarily parallel intracranial protection.

## Introduction

The incidence of melanoma has been on the rise worldwide, and it is a common cause of intracranial metastases [1]. The prevalence of intracranial metastases in melanoma can range from 10 to 40% during a patient’s lifetime [2-4]. The emergence of advanced BRAF/MEK targeted therapy and immunotherapy have resulted in significant improvement in survival rates among advanced-stage melanoma patients [5-7]. Stereotactic radiosurgery (SRS) is a common treatment for brain metastases, and many of the intracranial lesions treated by SRS at our facility are metastases lesions [8-11]. Recently, we have noticed a trend among patients with melanoma treated with SRS at our institution: many develop purely intracranial disease in the absence of extracranial disease or the presence of controlled extracranial disease. Proactively treating intracranial metastases not only leads to intracranial disease control but also provides survival benefits for patients with controlled extracranial disease at the time of development of brain metastasis [12,13]. The additional benefit from proactive SRS or surgery is maintained even in patients being treated with immunotherapy and targeted therapy [14].

In this study, we reviewed all cases of patients treated with SRS for melanoma metastases in an effort to quantify the degree to which BRAF/MEK targeted therapy and immunotherapy show an extracranial response with the development of intracranial metastatic diseases.

## Materials and methods

This study was approved by our institutional review board. It was a single-center, retrospective analysis of patients who received SRS as primary treatment for brain metastasis from metastatic melanoma. Subjects were compiled from the SRS database at our institution. Charts were accessed from the institution's electronic medical record system. All data was collected and de-identified prior to being reviewed by the authors. We calculated the data based on the date of the primary diagnosis of melanoma. All patients had resection of primary melanoma, allowing for the testing of BRAF mutation status of primary melanoma. We also analyzed the use of BRAF/MEK inhibitor therapy, immunotherapy, duration of systemic therapy before SRS, prior brain surgery, age at the time of SRS, the location and timing of extracranial disease burden, and whether extracranial disease burden worsened or improved on immune therapy.

## Results

A total of 51 patients underwent SRS for intracranial melanoma metastases during the study period. Of these patients, 36 were males, 15 were females, and they ranged in age from 29 to 88 years old with an average age of 58.6. Table 1 presents the patient demographics and tumor characteristics.

**Table 1:** Patient demographics and tumor characteristics *Did not undergo testing SRS: stereotactic radiosurgery

Characteristics of the study population (n=51)		
Patient sex		
Male	36 (70.6%)	
Female	15 (29.4%)	
BRAF status		
Positive	26 (51%)	
Negative	21 (41%)	
N/A*	4 (8%)	
Surgery prior to SRS		
Yes	17 (33.3%)	
No	34 (66.7%)	
Systemic disease present at the time of SRS		
Yes	42 (82.4%)	
No	9 (17.6%)	
Age at the time of SRS		
Average: 58.6 years		
Range: 29-88 years		
Primary tumor site		
Scalp/face		6
Neck		5
Chest		6
Back		9
Extremities		11
None identified		7

Among the patients, 51% were positive for BRAF, 41% were negative, and 8% did not undergo testing. At the time of SRS, 42 (82.4%) patients had extracranial disease in addition to their primary disease site, while nine (17.6%) did not. Of the patients with the extracranial disease, 34 received systemic therapy prior to receiving SRS. Eight of the 34 patients (23.5%) received only targeted therapy (BRAF/MEK inhibitors), 16 (47.1%) received only immunotherapy prior to SRS, and 10 (29.4%) received both targeted therapy and immunotherapy before SRS. A total of 34 patients were on immunotherapy or targeted therapy prior to receiving SRS. We then determined which patients had progression of the extracranial disease (Table 2).

**Table 2:** Extracranial disease progression based on the type of systemic therapy received

Extracranial disease progression		
Progression of extracranial disease on BRAF/MEK targeted therapy (n=8)	Yes	2 (25%)
	No	6 (75%)
Progression of extracranial disease on immunotherapy (n=16)	Yes	9 (56.25%)
	No	7 (43.75%)
Progression of extracranial disease on BRAF/MEK targeted therapy and immunotherapy (n=10)	Yes	7 (70%)
	No	3 (30%)
Overall progression of extracranial disease (n=34)	Yes	18 (52.9%)
	No	16 (47.1%)

## Discussion

Melanoma is known to have a potential for intracranial disease progression in the setting of positive extracranial response. This was initially reported pertaining to biomodulator therapies in the 1980s [15]. Recently, with the advent of BRAF/MEK inhibitors and immunologic therapies, the unmasking of intracranial metastatic disease from melanoma in the setting of systemic disease control has also been reported [16-22]. The presence of intracranial metastatic disease in melanoma is of diagnostic and prognostic importance. In fact, the most recent guidelines for melanoma staging from the American Joint Committee on Cancer have created the new category of “M1d” to specify the presence of central nervous system (CNS) metastatic disease burden [23].

The use of targeted and immunotherapies alone has not been able to provide an adequate response to intracranial metastases from melanoma. A recent phase 2 trial conducted in 2016 for pembrolizumab for patients with untreated brain metastases demonstrated 4/18 (22%) patients with melanoma to have some degree of response to treatment [24]. The CheckMate204 study (a phase 2 trial) demonstrated a 26% complete response rate, as well as a 30% partial response rate, among 96 patients in the post-treatment phase with both nivolumab and ipilimumab after a median follow-up of 14 months [25]. One study cites only an approximate 50% response rate to immunotherapy intracranially, and even so, the intracranial response is not as prolonged as the extracranial response [26]. There have been multiple trials evaluating the efficacy of nivolumab, ipilimumab, or pembrolizumab for intracranial disease, yet the CheckMate204 trial has demonstrated the best response rates for intracranial metastatic melanoma by far [27]. There is also literature supporting the idea that the addition of SRS to immunotherapy as an initial treatment for brain metastases from melanoma is effective [28].

However, these studies do not address patients diagnosed with melanoma initially without intracranial disease and who then develop new intracranial disease after being placed on targeted therapies or immunotherapies. The use of each individual immunotherapy for patients with primary melanoma disease with undiagnosed/undeveloped intracranial metastasis is largely dependent on the patient’s oncological provider, and the consideration of adverse side effects of the drug in the setting of a patient’s overall condition and comorbidities. Therefore, regardless of the efficacy of each individual therapy or combination of therapies in treating known metastases, this study serves an important role in quantifying the development of intracranial metastatic disease in patients who are already on the therapeutic regimens. Our rationale for attempting to quantify this association between extracranial response with intracranial disease progression is to raise the question as to whether or not aggressive monitoring for intracranial disease in patients on BRAF/MEK targeted and immunologic therapies is required.

Our results show that almost half (47.1%) of the patients treated with any combination of BRAF/MEK targeted therapy and immunotherapy develop intracranial disease in the setting of stable or improving extracranial disease, with only 52.9% having progressing extracranial disease at the time of clinical presentation. There is a difference in terms of survival in patients who have intracranial metastatic melanoma and lower functional status due to symptomatic disease compared to those with incidentally found disease [29]. In addition, the Graded Prognostic Assessment score for intracranial melanoma is lower, indicating lower median survival, for patients who are more severely symptomatic as evidenced by a lower Karnofsky Performance Scale [30]. It is therefore critical to understand and define the incidence of development of intracranial disease in patients on immunotherapies, and to our knowledge, our paper is the first of its kind to quantify this parameter in a patient population.

Due to our small patient population, we were unable to reveal whether a single therapy or combination of therapies is more effective at intracranial protection. Also, since our study was retrospective and not prospective, we could not control for the decision as to why each patient received the treatment regimens they did, and in what order or combination. Overall, our data reveal that the patients who received both BRAF/MEK targeted therapy and immunotherapy show the highest degree of extracranial disease progression, at 70%. This could be due to selection bias as ours was a population that was already selected for a more aggressive clinical course, and hence the requirement for two regimens attempted in the treatment course. Regardless, our study suggests that there is a significant incidence of intracranial disease burden from melanoma even in the setting of controlled or improving extracranial disease in patients while on BRAF/MEK targeted therapy and immunotherapies.

## Conclusions

Sixteen patients (47.1%) developed intracranial metastasis in our study while having stable systemic disease. This was observed in patients receiving BRAF/MEK targeted therapy, immunotherapy, or a combination of the two. This finding has ramifications for intracranial screening for patients with melanoma. Close intracranial monitoring and imaging follow-up should be conducted in metastatic melanoma patients on immunotherapy or a BRAF/MEK combination, even if they are asymptomatic.

Our data suggest that systemic response to therapy does not indicate intracranial protection. We have quantified the magnitude of this relationship for the first time and hopefully laid the groundwork for further studies. Larger studies will help us to better analyze the actual differences in the degree of intracranial protection between each of the treatments or combinations of treatments with more statistical certainty.
